# Characterization of the symptoms of neurogenic orthostatic hypotension and their impact from a survey of patients and caregivers

**DOI:** 10.1186/s12883-018-1129-x

**Published:** 2018-08-25

**Authors:** Daniel O. Claassen, Charles H. Adler, L. Arthur Hewitt, Christopher Gibbons

**Affiliations:** 1 Department of Neurology, Vanderbilt University Medical Center, 1161 21st Avenue South A-0118, Nashville, TN 37232 USA; 2 Parkinson’s Disease and Movement Disorders Center, Department of Neurology, Mayo Clinic College of Medicine, Mayo Clinic, 13400 East Shea Boulevard, Scottsdale, AZ 85259 USA; 3 Medical Affairs, Lundbeck, 6 Parkway North, Deerfield, IL 60015 USA; 4 Department of Neurology, Beth Israel Deaconess Medical Center, Harvard Medical School, 330 Brookline Avenue, Boston, MA 02215 USA

**Keywords:** Neurogenic orthostatic hypotension, Parkinson disease, Multiple system atrophy, Quality of life, Disease burden

## Abstract

**Background:**

Neurogenic orthostatic hypotension (nOH) results from impaired vasoconstriction due to dysfunction of the autonomic nervous system and is commonly associated with Parkinson disease (PD), multiple system atrophy (MSA), and pure autonomic failure. nOH can increase the risk of falls due to symptoms that include postural lightheadedness or dizziness, presyncope, and syncope. The purpose of this study was to obtain information from patients and caregivers regarding the symptoms and burden of nOH to expand on limited knowledge regarding the impact of nOH on quality of life.

**Methods:**

This author-designed survey included questions regarding nOH (e.g., frequency and impact of symptoms, management) and was conducted online by Harris Poll via distribution to individuals who agreed to participate in Harris Poll online surveys or who were members of relevant disease advocacy organizations. Eligible patients were aged ≥ 18 years with PD, MSA, or pure autonomic failure and ≥ 1 of the following: orthostatic hypotension (OH), nOH, low blood pressure upon standing, or OH/nOH symptoms. Eligible caregivers cared for such patients but were not necessarily linked to any patient participant.

**Results:**

Survey responses were received from 363 patients and 128 caregivers. PD was the most frequent underlying disorder (90% of patients; 88% of individuals managed by the caregivers). Despite meeting survey diagnosis criteria, a formal diagnosis of OH or nOH was reported by only 36% of patients and 16% of caregivers. The most frequent symptoms of nOH were dizziness or lightheadedness, fatigue when standing, and difficulty walking. A negative impact on patient quality of life caused by nOH symptoms was reported by 59% of patients and 75% of caregivers. Most respondents (≥87%) reported that nOH symptoms adversely affected patients’ ability to perform everyday activities (most frequently physical activity/exercise, housework, and traveling). Falls (≥1) in the previous year due to nOH symptoms were reported by 57% of patients and 80% of caregivers.

**Conclusions:**

These survey results support the premise that nOH symptoms have a substantial negative impact on patient function and quality of life. The relatively low rates of formal nOH/OH diagnosis suggest the need for heightened awareness regarding the condition and its symptom burden.

**Electronic supplementary material:**

The online version of this article (10.1186/s12883-018-1129-x) contains supplementary material, which is available to authorized users.

## Background

Orthostatic hypotension (OH) is defined as a sustained reduction in systolic blood pressure (BP) of ≥20 mmHg or in diastolic BP of ≥10 mmHg upon standing [1]. OH generally results from 3 common etiologies: (1) medications such as antidepressants or antihypertensive agents, (2) non-neurologic conditions such as hypovolemia or cardiovascular disorders causing cardiac failure, or (3) impaired vasoconstriction due to dysfunction of the autonomic nervous system (also referred to as neurogenic OH [nOH]). The neurogenic form of OH is commonly associated with neurodegenerative disorders that affect the central or peripheral autonomic nervous system, such as Parkinson disease (PD), multiple system atrophy (MSA), and pure autonomic failure, or it may be secondary to conditions such as diabetic peripheral neuropathy [1–6]. Although differential diagnosis is often challenging, nOH can be distinguished from non-neurogenic forms of OH, such as medication effects and volume depletion, through autonomic testing [5, 7]. Common symptoms of nOH include postural lightheadedness or dizziness, presyncope, falls, and syncope [1, 5]. Additional symptoms can include visual disturbances, fatigue, generalized weakness, cognitive dysfunction, neck pain or discomfort in the suboccipital and paracervical region (i.e., in a “coat hanger” configuration), and orthostatic dyspnea [1, 5].

Neurogenic OH can increase the risk of falls, particularly among older patients [8, 9]. However, only limited information regarding the impact of nOH on quality of life among patients and caregivers has been published [10–12]. We designed a survey to gain a better understanding of the following areas: (1) scope of symptoms and burden of disease among patients with nOH, (2) effect of nOH symptoms on lives of patients from the perspective of caregivers, and (3) insights on the patient and caregiver journey from diagnosis to symptom management.

## Methods

A survey designed by the authors was conducted online by Harris Poll on behalf of Lundbeck between August 26, 2016, and October 3, 2016. Respondents for the survey included individuals who agreed to participate in Harris Poll online surveys or who are members of certain advocacy organizations (American Parkinson Disease Association, Davis Phinney Foundation, Michael J. Fox Foundation, MSA Coalition, National Parkinson Foundation, and Parkinson’s Disease Foundation) who also met eligibility criteria and agreed to participate in the current survey. For research in which participants are intended to remain anonymous, Harris Poll uses tools and methods to ensure that there is no reasonable possibility of identifying an individual participant in the reports created (e.g., individual responses collected are combined to produce “aggregated” reports). Eligible patient participants were US residents aged ≥18 years who self-selected a diagnosis of PD, MSA, or pure autonomic failure based on a diagnosis received from their treating physicians. Individuals also met ≥1 of the following criteria: (1) received a formal diagnosis of OH or nOH, (2) were informed by a health care provider that their symptoms are caused by low blood pressure or a sudden drop in blood pressure upon standing, or (3) experience the following upon sitting up, standing up, standing for long periods of time, or with a change in position: 2 or more listed OH/nOH symptoms at least every time, daily, weekly, monthly, or a few times a year and at least 1 of the following symptoms: dizziness or lightheadedness, feeling faint, or fainting. Because of the underlying neurologic diagnosis criteria, eligible patient responders were presumed to have "nOH" for the purposes of this study, even if they did not receive a formal diagnosis. Eligible caregiver participants cared for patients who met these criteria but were not necessarily linked to any patient responders (i.e., patient and caregiver responses to surveys were not paired in the analysis of survey results). The survey included questions regarding the frequency and impact of nOH symptoms, management, and communication with health care providers regarding symptoms (see Additional File 1 for the full list of survey questions). Descriptive statistics are reported. The study was performed in accordance with ethical standards (e.g., 1964 Helsinki Declaration and later amendments or the comparable). As an anonymous survey, the study was exempt from ethics approval based on Code of Federal Regulations Title 45, Part 46, Subpart A, Section 46.101b, Category 2 criteria.

## Results

### Respondents

Demographic data are provided in Table 1. A total of 363 patients (mean age ± standard deviation, 63.4 ± 12.4 years) and 128 caregivers responded to the survey. Among the caregivers, the mean ± standard deviation age of the patient cared for was 70.7 ± 14.8 years; 46% provided care to a spouse or partner. Most patients experienced long-term nOH symptoms, with 48% and 21% of patient respondents reporting living with symptoms for ≥5 and ≥ 10 years, respectively (mean ± standard deviation, 7.8 ± 10.0 years). Among caregivers, 59% and 38% reported that the patient cared for lived with symptoms for ≥5 and ≥ 10 years. PD was the most frequent underlying disorder, identified by 90% of patients and 88% of caregivers reported providing care to patients with PD. A formal diagnosis of OH or nOH was reported by 36% of patients and by 16% of caregivers. A longer duration of symptoms did not increase the proportion of patients with a formal diagnosis of OH or nOH; among patients with a symptom duration of ≥10 years compared with < 10 years, 35% (27/78) versus 36% (102/285) reported a formal diagnosis of one of these conditions.

**Table 1:** Baseline Characteristics of Survey Respondents

Characteristic	Patients (*n* = 363)	Caregivers (*n* = 128)	
		Self	Patient Being Cared for
Men	51%	37%	54%
Mean ± SD age, y	63.4 ± 12.4	56.2 ± 14.9	70.7 ± 14.8
White	90%	92%	NA
Neurologic diagnosis^a^			
Parkinson disease	90%	NA	88%
Multiple system atrophy	10%	NA	11%
Pure autonomic failure	4%	NA	3%
Mean ± SD years experiencing nOH symptoms	7.8 ± 10.0	NA	10.0 ± 9.9
Years living with nOH symptoms			
< 1	10%	NA	5%
1–4	42%	NA	37%
5–9	27%	NA	21%
≥ 10	21%	NA	38%
Formal diagnosis of nOH or OH	36%	NA	16%

*NA* not available, *nOH* neurogenic orthostatic hypotension, *OH* orthostatic hypotension

^a^Multiple responses could be selected; therefore, the sum of percentages is > 100%

Among all respondents, 49% of patients reported that they were in fair or poor health, with 72% of caregivers reporting that the patients they provided care for were in fair or poor health. Among the subgroup with a diagnosis of PD or MSA, 34% of patients (121/357) and 49% of caregivers (61/125) somewhat or strongly agreed that nOH symptoms appeared before patients developed motor symptoms. Among patients diagnosed with PD (*n* = 328), 44% somewhat or strongly agreed that their nOH symptoms were more troublesome than their motor symptoms. Findings from caregivers of patients with PD (*n* = 113) were similar (47%).

### nOH symptom experience and impact

#### nOH symptoms

**Fig. 1:**
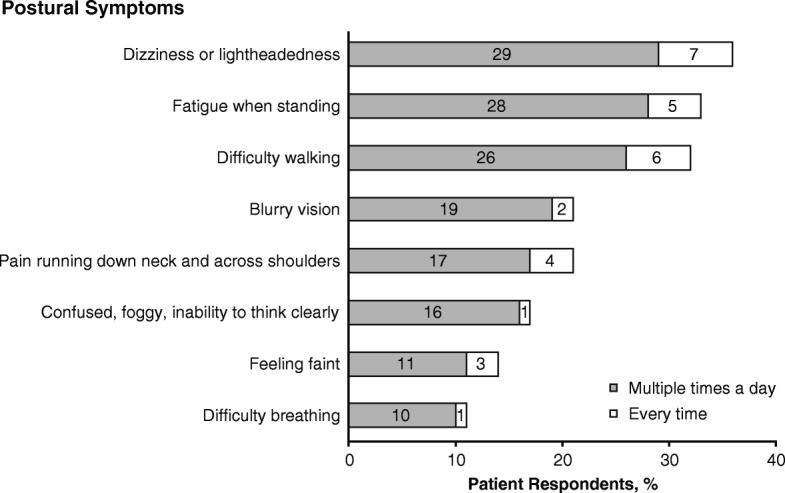
Proportion of patient respondents reporting postural nOH symptoms.* nOH=neurogenic orthostatic hypotension. *Reported symptoms could be experienced upon sitting or standing up, when standing for long periods of time, or during a change in position

The most frequently reported symptoms of nOH were dizziness or lightheadedness, fatigue when standing, and difficulty walking; a substantial proportion of patients reported that dizziness or lightheadedness (37%), fatigue when standing (33%), and difficulty walking (32%) occurred every time or multiple times a day when they sit up, stand up, are standing for long periods of time, or have a change in position (Fig. 1). Other symptoms reported as occurring multiple times a day by **>** 10% of patients included blurry vision, pain running down neck and across shoulders, cognitive difficulties, faintness, and difficulty breathing (Fig. 1).

**Fig. 2:**
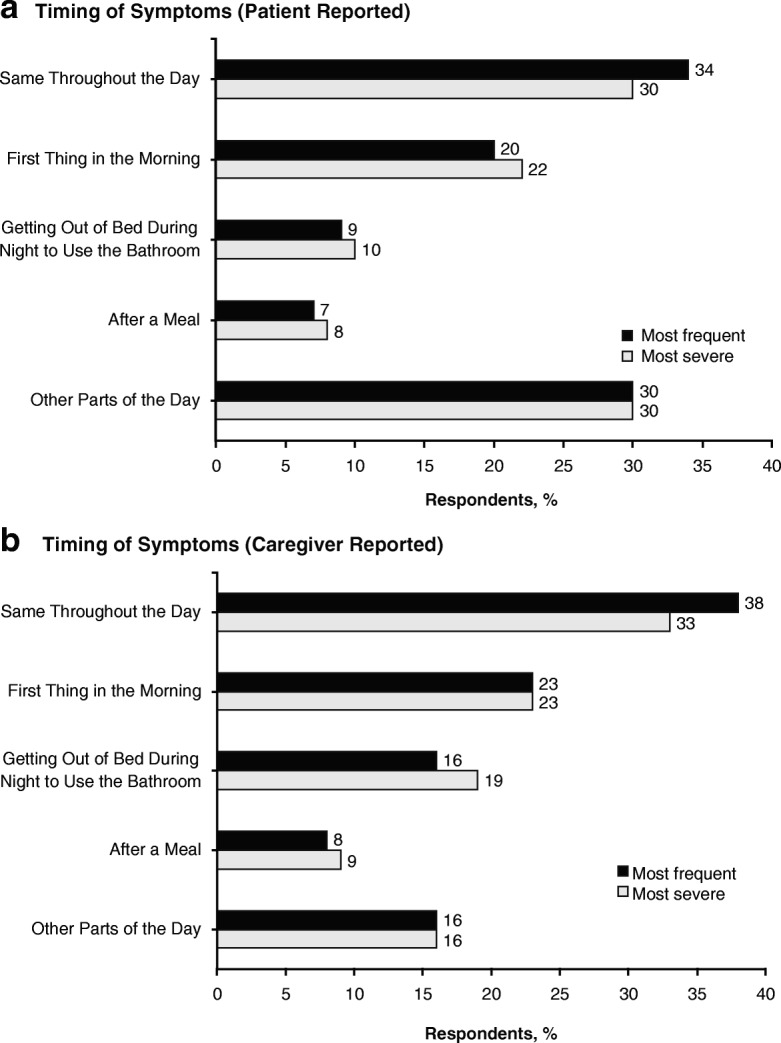
Daily pattern of most frequent/severe nOH symptoms as reported by **a** patients and **b** caregivers.* nOH = neurogenic orthostatic hypotension. *Respondents in the patient and caregiver cohorts were not paired

No distinct pattern emerged regarding the frequency and severity of nOH symptoms throughout the day **(**Fig. 2**)**. When asked about conditions that exacerbated their nOH symptoms, the majority of patients and caregivers (61% each) somewhat or strongly agreed that nOH symptoms worsened in hot and/or humid conditions. Exacerbation of nOH symptoms after meals was reported less frequently (27% of patients, 34% of caregivers). Falls due to nOH symptoms (at least 1 in the previous year) were reported by 57% of patients (mean, 5.1 falls) and 80% of caregivers (mean, 7.8 falls).

#### Functional impact of nOH symptoms

**Fig. 3:**
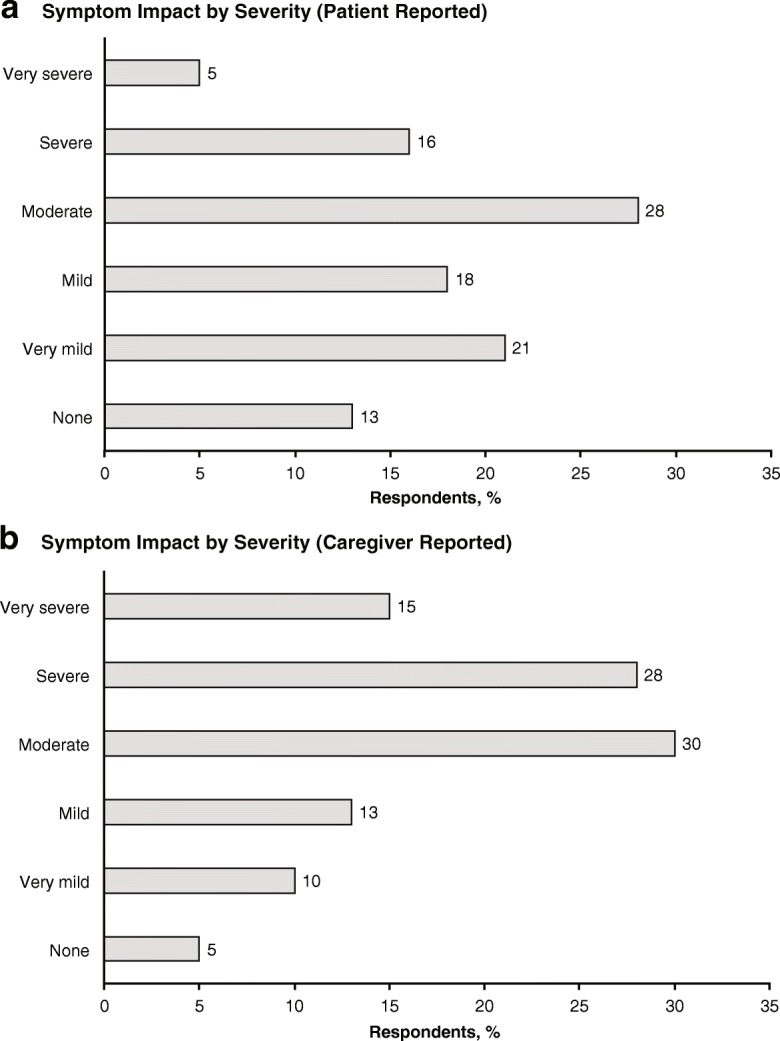
nOH symptom impact on patient daily activities as reported by **a** patients and **b** caregivers.*^,†^ nOH = neurogenic orthostatic hypotension. *Respondents in the patient and caregiver cohorts were not paired. ^†^Percentages rounded to the nearest whole number

The majority of patients (87%) and caregivers (95%) reported that nOH symptoms had an overall negative impact on patients’ ability to perform everyday activities; this was categorized as severe or very severe by approximately one-fifth of patients and two-fifths of caregivers **(**Fig. 3**)**. A substantial proportion of patients reported that nOH symptoms negatively impacted their quality of life (59%), robbed them of their independence (42%), or drastically changed their life (40%). In the caregiver cohort, 75% of respondents reported that nOH symptoms had a negative impact on the patient’s quality of life, and approximately two-thirds reported that nOH symptoms had robbed patients of their independence (66%) or drastically changed their life (65%). The symptom burden of nOH did not appear to be affected by the duration of symptom experience. Similar proportions of patients with nOH symptoms for < 10 years and ≥ 10 years reported that symptoms had a negative impact on their quality of life (60% and 55%, respectively), caused a drastic change in their life (38% and 49%), or robbed them of their independence (41% and 47%).

**Fig. 4:**
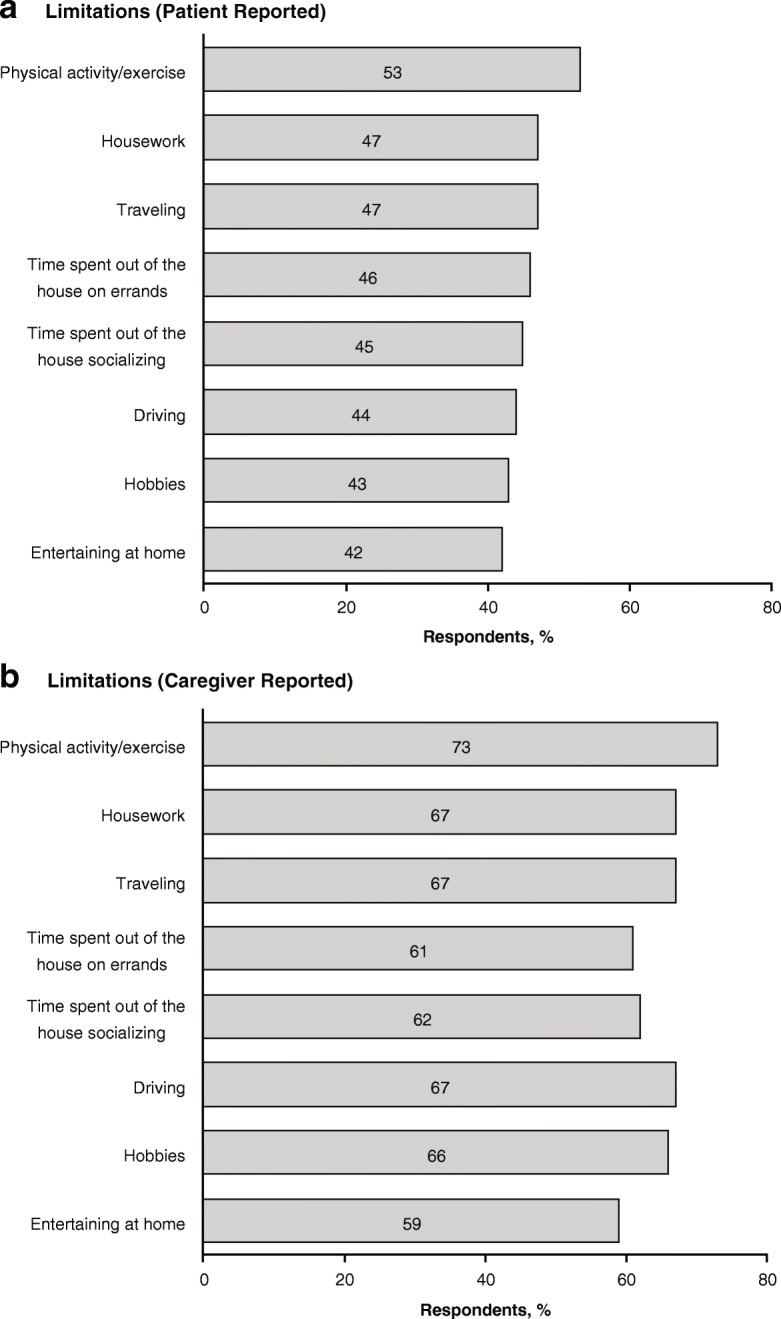
nOH symptom-related limitations on patient daily activities as reported by **a** patients and **b** caregivers.* nOH = neurogenic orthostatic hypotension. *Percentages represent the proportion of respondents who reported the patient reduction or stopping of the activity. Respondents in the patient and caregiver cohorts were not paired

Activities that were reported as reduced or stopped because of symptoms of nOH by > 40% of patients were physical activity/exercise, housework, traveling, time spent out of the house to run errands or socialize, driving, hobbies, and entertaining at home **(**Fig. 4a**)**. Caregivers reported that such activities were reduced or stopped in ≥59% of the patients they care for **(**Fig. 4b**)**. More than half (56%) of patients and 85% of caregivers reported that patients needed assistance with day-to-day activities (e.g., walking, getting out of a chair) in the past month, and up to 53% of patients and 73% of caregivers reported that some daily activities were reduced or stopped because of nOH symptoms.

Half of patients (50%) somewhat or strongly agreed that nOH symptoms caused them anxiety or worry and somewhat or strongly agreed that the management of symptoms caused them to be depressed or discouraged; 39% somewhat or strongly agreed that they really struggled to get their nOH symptoms under control. Despite the negative impact of nOH symptoms on functionality and quality of life, 60% of patients and 53% of caregivers somewhat or strongly agreed that patients often hide or minimize their nOH symptoms.

#### Patient/health care provider interactions and the path to diagnosis of nOH

Most patients (75%) and caregivers (77%) somewhat or strongly agreed that they were satisfied with the quality of communication with health care providers. The health care providers most commonly seen (i.e., reported by > 20% of patients or caregivers) for the management of underlying medical conditions manifesting with nOH were primary care providers, movement disorder specialists, general neurologists, and general cardiologists. A delay of 6 months or more between the time of symptom onset to discussion with a health care provider was reported by 33% of patients and 41% of caregivers. Patients and caregivers (55% each) somewhat or strongly agreed that patients did not initiate a discussion about their nOH symptoms with their health care provider unless the symptoms were severe, and 26% of patients and 39% of caregivers somewhat or strongly agreed that patients were uncomfortable talking with their health care provider about the impact of nOH symptoms.

Approximately one-fourth (26%) of patients and a third (33%) of caregivers somewhat or strongly agreed that patients had to mention their nOH symptoms repeatedly to their health care provider to draw attention to the problem. In the subgroup of 129 patients with a formal diagnosis of nOH or OH, 43% reported seeing 3 or more health care providers before being diagnosed with OH or nOH. Half (50%) of the patients who were formally diagnosed somewhat or strongly agreed that the path to diagnosis of nOH was very frustrating. However, 70% of the patients formally diagnosed with OH/nOH somewhat or strongly agreed that management of their symptoms improved after diagnosis. Only 20 caregiver responders cared for patients who had a formal diagnosis of OH/nOH, so a similar subgroup analysis was not performed.

#### Symptom management and treatments/interventions

Approximately half (53%) of patients and 62% of caregivers somewhat or strongly agreed that they received a solution from health care providers to better manage symptoms of nOH. Interventions recommended by health care providers were avoidance of quick positional changes, increased fluid intake, adjustment of PD medications, increased salt intake, use of compression garments, elevating the head of the bed, and avoiding hot environments. Caregivers reported the same interventions with similar frequency **(**Table 2**)**. A third (34%) of patients and 45% of caregivers reported that a prescription for a medication to treat nOH symptoms was received; 25% of patients and 9% of caregivers reported that patients were not counseled to do anything to manage their nOH symptoms.

**Table 2:** Recommended Interventions for nOH by Health Care Providers

Intervention	Patients Reporting (n = 363)	Caregivers Reporting (n = 128)
Avoid quick positional changes	49%	48%
Increase fluid intake	47%	52%
Adjust PD medication	28%	43%
Increase salt intake	27%	20%
Wear compression stockings and/or abdominal binders	24%	27%
Elevate head of the bed	22%	28%
Avoid heated environments	16%	16%
Adjust or discontinue blood pressure or heart medications	15%	28%
Physical counter maneuvers (e.g., standing up and crossing legs, standing up and squeezing hands tightly)	12%	13%
No intervention recommended	25%	9%

*nOH* neurogenic orthostatic hypotension, *PD* Parkinson disease

## Discussion

The results of this patient and caregiver survey strongly support the premise that nOH symptoms have a substantial negative impact on patient function and quality of life. Data from both patients and caregivers suggest that nOH symptoms are associated with impaired mobility, such as difficulty with positional changes, increased frequency of falls, and decreased ability to maintain activities of daily life. Despite the high level of symptom burden and longstanding symptoms of nOH (e.g., 48% of patients with symptoms for ≥5 years), the majority of respondents did not report having a formal diagnosis of OH or nOH. Stratification of patients based on their duration of symptoms (< 10 years or ≥ 10 years) did not increase the likelihood of a formal diagnosis of OH or nOH. Further, there were minimal differences on the impact of nOH symptoms between these 2 subgroups of patients.

In this survey, the majorities of patients and caregivers did not report that nOH symptoms occurred more frequently or with greater severity first thing in the morning, after meals, or when getting out of bed during the night. Rather, more respondents indicated that nOH symptoms were more likely to occur or be more severe throughout the day. These results are contrary to the general clinical understanding that nOH symptoms are often worse in the morning and can be exacerbated after eating [13]. The lack of a distinct daily pattern for the frequency and severity of nOH symptoms emphasizes that symptoms do not always occur at a specific time or event (e.g., morning, after a meal) and is an important point of awareness for clinicians in the evaluation of patients for nOH.

This survey also revealed factors that may contribute to the relatively low rate of formal nOH diagnosis. The majority of patients reported hiding or minimizing their nOH symptoms. Further, patients indicated that they were uncomfortable discussing the impact of symptoms with health care providers and did not discuss the impact of their symptoms unless they were severe. Finally, some patients and caregivers reported that symptoms had to be discussed several times in order to draw them to the attention of the treating provider.

Reticence of patients to share symptoms with caregivers and health care providers has been observed with depression, pain, and other chronic conditions [14–16]. Patients may hide or minimize their nOH symptoms because they view symptoms as a sign of weakness or because they are embarrassed, in denial, do not want to be a “bother” to others, or other reasons [14–16]. Of note, among patients in the current survey with a diagnosis of nOH, many (70%) perceived that their symptoms had improved after receiving the diagnosis. However, a substantial proportion of patients were not counseled on how to manage nOH, were not prescribed medication for nOH symptoms, and felt they did not have adequate control of their nOH symptoms.

Inadequate management of nOH symptoms appears to be a cause of distress for many patients. Nonpharmaceutical treatment recommendations for nOH include ensuring adequate salt and fluid intake, avoiding rapid postural changes, adjusting medications, and sleeping with the head of the bed elevated [5, 17]. Patients and caregivers indicated that such nonpharmaceutical interventions were commonly recommended and frequently helpful. However, a substantial proportion of respondents indicated that they did not achieve symptomatic relief, and only a third of patients indicated that they received a prescription for a medication to treat symptoms of nOH. These results provide greater understanding of the disease burden of nOH. Previously, a negative impact of nOH symptoms was found in a single-center study of 141 inpatients with PD, in which 53% of patients reported that orthostatic dizziness had “a lot” or “very much” impact on daily life [11]. A 2-center study of patients with PD has suggested that both symptomatic (*n* = 14) and asymptomatic (*n* = 23) nOH were associated with worse measures of functionality and quality of life compared with patients with PD but without nOH (*n* = 84) [18]. Our study adds to the evidence by inclusion of data from a larger cohort of patients (*N* = 363) who experience nOH symptoms due to a variety of underlying conditions, including MSA and pure autonomic failure (however, most of the cohort reported a PD diagnosis). Further, to the best of our knowledge, our study is the first to investigate the impact of nOH from both the patient and caregiver perspective. nOH is one of the many manifestations of autonomic dysfunction, and patients may also have their quality of life affected by other symptoms, including gastrointestinal dysfunction (e.g., dysphagia, constipation), bladder dysfunction (e.g., urinary urgency, incontinence), sexual dysfunction (e.g., erectile dysfunction), cardiovascular dysfunction (e.g., hypertension, supine hypertension), and thermoregulation and sweating abnormalities (e.g., dyshidrosis) [19, 20].

Survey methodology has inherent limitations, such as selection bias and recall bias. Patient and caregiver responses were not paired; therefore, it is not possible to draw conclusions regarding consistency of responses between patients and their caregivers. Pairing each patient’s response with their caregiver’s response for each question should be considered in future studies. Many participants (64% of patients, 84% of caregivers) did not have a formal diagnosis of OH or nOH; therefore, the survey may underestimate the burden of symptoms of those formally diagnosed with OH or nOH.

## Conclusions

The findings from this survey underscore a significant symptom burden associated with nOH. Many patients have a delay or lack of diagnosis of nOH, which creates diagnostic uncertainty and slows symptom management. As a consequence of this under-recognition of nOH, patients may face an increased risk of falls and associated morbidity [21, 22]. This study highlights the need for a more timely diagnosis of nOH. Improved patient/provider communication about symptoms of nOH may facilitate more timely and appropriate intervention for these patients. Overall, heightened awareness regarding nOH and its symptom burden should be an educational priority for patients, as well as for their caregivers and for health care providers.

## Additional file

Additional file 1: Online Survey for Patient and Caregiver Characterization of Neurogenic Orthostatic Hypotension Symptoms and Impact (DOCX 60 kb)

## Abbreviations

BP: blood pressure
MSA: multiple system atrophy
NA: not available
nOH: neurogenic orthostatic hypotension
OH: orthostatic hypotension
PD: Parkinson disease
