# Supplementary material for: Characterization of the symptoms of neurogenic orthostatic hypotension and their impact from a survey of patients and caregivers
Source: BMC Neurol. 2018 Aug 25;18:125. doi: 10.1186/s12883-018-1129-x (PMC6109309; doi:10.1186/s12883-018-1129-x)
Supplement: Supplementary file 1 — Online Survey for Patient and Caregiver Characterization of Neurogenic Orthostatic Hypotension Symptoms and Impact (DOCX 60 kb) [file 12883_2018_1129_MOESM1_ESM.docx]

**Additional File 1**

**Online Survey for Patient and Caregiver Characterization of Neurogenic Orthostatic Hypotension Symptoms and Impact**

This document includes the full list of questions in the survey administered to patients and caregivers. The survey was designed by the authors and conducted online by Harris Poll on behalf of Lundbeck. Only a portion of patient/caregiver responses are described in the accompanying manuscript titled “Characterization of the Symptoms of Neurogenic Orthostatic Hypotension and Their Impact From a Survey of Patients and Caregivers.”

**SURVEY**

**Duration:** Approximately 20 minutes

**Questions:**

ALL RESPONDENTS

ALL RESPONDENTS

Q1000 PRELOAD: Organizations

1. Harris Poll Online
2. The Michael J Fox Foundation (MJFF)
3. National Parkinson's Foundation (NPF)
4. Parkinson's Disease Foundation (PDF)
5. MSA Coalition
6. Davis Phinney Foundation
7. American Parkinson's Disease Association (APDA)\

Section 100: Screener

ALL RESPONDENTS

Q258 (Q700) In which country or region do you currently reside?

14 Australia

15 Austria

24 Belgium

42 Canada

60 Denmark

76 France

85 Germany

89 Greece

123 Italy

168 Netherlands

171 New Zealand

179 Norway

190 Portugal

215 Spain

223 Sweden

224 Switzerland

244 United States of America

266 England

267 Scotland

268 Wales

285 Northern Ireland

120 Ireland (Republic of Ireland)

996 Other country

ALL RESPONDENTS

Q268 (Q705) Are you…?

1. Male
2. Female

ALL RESPONDENTS

Q271 (Q710) In what month were you born?

1. January
2. February
3. March
4. April
5. May
6. June
7. July
8. August
9. September
10. October
11. November
12. December

BASE: ALL RESPONDENTS

Q270 (Q711) In what year were you born? Please enter your response as a four-digit number (for example, 1977).

[RANGE: 1900 TO CURRENT YEAR]

ALL RESPONDENTS

Q715 Next, we have a few employment and economic questions.

1. Yes
2. No
3. Are you employed full time for pay with an organization or company?
4. Are you employed part time for pay with an organization or company?
5. Are you self-employed full time?
6. Are you self-employed part time?

NOT AT ALL EMPLOYED

Q720 Which of the following best describes your current situation?

1. Looking for work
2. Not looking for work
3. Retired
4. Unable to work due to a disability or illness

ALL RESPONDENTS

Q730 Do any of the following describe you? [MULTIPLE RESPONSE]

1. Retired and collecting retirement benefits/pension
2. A student
3. A stay-at-home spouse or partner
4. None of these [EXCLUSIVE]

ALL RESPONDENTS

Q105 Overall, how would you describe your general health?

1. Poor
2. Fair
3. Good
4. Excellent

ALL RESPONDENTS

Q110 Have you ever been diagnosed with any of the following conditions?

1. Yes
2. No
3. Parkinson’s disease (PD) [QUALIFIED]
4. Multiple system atrophy (MSA) [QUALIFIED]
5. Pure autonomic failure (PAF) [QUALIFIED]
6. Orthostatic hypotension (OH) – a drop in blood pressure upon standing [QUALIFIED]
7. Neurogenic orthostatic hypotension (nOH) [QUALIFIED]
8. POTS (Postural Orthostatic Tachycardia Syndrome) [NOT ELIGIBLE]
9. Progressive supranuclear palsy (PSP) [NOT ELIGIBLE]
10. Dementia with Lewy bodies
11. Dementia related to another neurologic process
12. Alzheimer's disease
13. Mild cognitive impairment

ALL RESPONDENTS WHO DO NOT HAVE A CONDITION AT Q110 (Q110/NO FOR ALL CODES)

Q110B Do you currently provide routine, unpaid care to a friend or family member diagnosed with any of the following health conditions? If you provide care for more than one person, please answer for the person you provide the most amount of care for.

1. Yes
2. No
3. Parkinson’s disease (PD) [QUALIFIED]
4. Multiple system atrophy (MSA) [QUALIFIED]
5. Pure autonomic failure (PAF) [QUALIFIED]
6. Orthostatic hypotension (OH) – a drop in blood pressure upon standing [QUALIFIED]
7. Neurogenic orthostatic hypotension (nOH)[QUALIFIED]
8. POTS (Postural Orthostatic Tachycardia Syndrome) [NOT ELIGIBLE]
9. Progressive supranuclear palsy (PSP) [NOT ELIGIBLE]
10. Dementia with Lewy bodies
11. Dementia related to another neurologic process
12. Alzheimer's disease
13. Mild cognitive impairment

CARES FOR SOMEONE WHO HAS BEEN DIAGNOSED WITH PARKINSON’S, MSA, PAF, OH, OR NOH IN Q110 AND DO NOT HAVE A CONDITION AT Q110 (Q110/NO FOR ALL CODES)

Q111 Is the person you care for…

1. Male
2. Female

CARES FOR SOMEONE WHO HAS BEEN DIAGNOSED WITH PARKINSON’S, MSA, PAF, OH, OR NOH IN Q110 AND DO NOT HAVE A CONDITION AT Q110 (Q110/NO FOR ALL CODES)

Q112 How old is the person for whom you provide care? If you are not sure, please provide your best estimate.

|_|_| [RANGE 1-99] years old

CARES FOR SOMEONE WHO HAS BEEN DIAGNOSED WITH PARKINSON’S, MSA, PAF, OH, OR NOH IN Q110 AND DO NOT HAVE A CONDITION AT Q110 (Q110/NO FOR ALL CODES)

Q620 Is the person with [INSERT SELECTIONS FROM Q110] whom you care for your…?

- 1. Spouse/partner
  2. Parent
  3. Sibling
  4. Grandparent
  5. Other family member
  6. Friend
  7. Other

HAVE BEEN OR CARES FOR SOMEONE WHO HAS BEEN DIAGNOSED WITH PARKINSON’S, MSA, PAF, OH, OR NOH IN Q110

Q115 [PATIENT: In what year were you diagnosed with the following condition(s)? Your best estimate is fine.]

[CAREGIVER: In what year was the person you care for diagnosed with the following condition(s)? Your best estimate is fine.]

1. Parkinson’s disease (PD)
2. Multiple system atrophy (MSA)
3. Pure autonomic failure (PAF)
4. Orthostatic hypotension (OH) – a drop in blood pressure upon standing
5. Neurogenic orthostatic hypotension (nOH)

Q120 [PATIENTS: About how often do you experience the following symptoms when you sit up, stand up, are standing for long periods of time or have a change in position (i.e., sitting to standing)?]

[CAREGIVERS: In your opinion, about how often does the person you care for experience the following symptoms when he/she sits up, stands up, stands for long periods of time or has a change in position (i.e., sitting to standing)?]

1. Dizziness or lightheadednes
2. Passing out or fainting
3. Falling
4. Blurry vision
5. Tunnel vision
6. Fatigue when standing
7. Pain running down neck and across shoulders
8. Nausea
9. Difficulty breathing
10. Difficulty walking (e.g., Leg weakness or feeling like their legs will buckle )
11. Confused, foggy, inability to think clearly
12. [PATIENT: Blood pressure drops when you stand]
     [CAREGIVERS: Blood pressure drops when he/she stands]
13. Feeling faint
14. [PATIENT: Every time I change positions (e.g. lying to sitting or sitting to standing)]

[CAREGIVER: Every time they change positions (e.g. lying to sitting or sitting to standing)]

1. Multiple times a day
2. Once a day
3. Multiple times a week
4. Once a week
5. A few times a month
6. Once a month

9 A few times a year

1. Never/Not at all

EXPERIENCE AT LEAST ONE SYMPTOM A FEW TIMES A YEAR OR MORE FREQUENTLY IN Q120 (Q120/1,3-14,15 AND A FEW TIMES A YEAR OR MORE FREQUENTLY (1-7, 9 IN THE SCALE))

Q125 [PATIENT: You mentioned you have:]

[CAREGIVER: You mention the person you care for has:]

[DISPLAY SYMPTOMS EXPERIENCED 1-7, 9 IN THE SCALE IN Q120]

[PATIENT: Have you ever been told by a health care provider that low blood pressure or blood pressure dropping is the cause of your symptoms when you sit up, stand up, are standing for long periods of time or have a change in position (i.e., sitting to standing)?]

[CAREGIVER: Has the person you care for ever been told by a health care provider that low blood pressure or blood pressure dropping is the cause of his/her symptoms when he/she sits up, stands up, stands for long periods of time or has a change in position (i.e., sitting to standing)?]

1. Yes
2. No
3. Not sure

Qualification Criteria

- Living in the US
- Age 18+
- Formally diagnosed or cares for someone formally diagnosed with Parkinson's disease, MSA, PAF (Q110/1-3 YES OR Q110B/1-3 YES)
- Not diagnosed with POTS (Postural Orthostatic Tachycardia Syndrome) or Progressive supranuclear palsy (PSP) (Q111/6 OR 7 NO)
- One of the following:
  - Formally diagnosed or cares for someone formally diagnosed with OH (Q110/4 YES OR Q110B/4 YES)
  - Formally diagnosed or cares for someone formally diagnosed with nOH (Q110/5 YES OR Q110B/5 YES)
  - Has been told by an HCP or cares for someone that has been told by an HCP that their symptoms at Q120 are caused by low blood pressure or blood pressure dropping upon standing (Q125/1)
  - Patients who or cares for someone who experience 2 or more listed OH/nOH symptoms at least every time, daily, or weekly, monthly, or a few times a year when they sit up, stand up, are standing for long periods of time or have a change in position (i.e., sitting to standing) AND at least one of the following: dizziness or lightheadedness, feeling faint, OR passing out or fainting (One of Q120/1, 3, 15 and at least 2 total symptoms)

ALL QUALIFIED RESPONDENTS

Q130 Before taking this survey, have you ever heard of the following conditions?

1. Never heard of
2. Know the name only
3. Somewhat familiar
4. Very familiar

[SHOW IN SAME ORDER AS IN Q110]

1. Orthostatic Hypotension (OH)
2. Neurogenic Orthostatic Hypotension (nOH)

Section 200: Experience with Symptoms

ALL QUALIFIED RESPONDENTS

Q200 [PATIENT: Throughout the remainder of the survey, we will focus on your experience with [IF FORMALLY DIAGNOSED WITH OH OR NOH IN Q110, DISPLAY: orthostatic hypotension (OH). In this survey, we will refer to it as nOH] [IF FORMALLY DIAGNOSED WITH OH NOH IN Q110, DISPLAY: neurogenic orthostatic hypotension (nOH)]. When we ask about symptoms, please only think about your symptoms related to [IF FORMALLY DIAGNOSED WITH OH IN Q110, DISPLAY: orthostatic hypotension (OH)] [IF FORMALLY DIAGNOSED WITH NOH IN Q110, DISPLAY: neurogenic orthostatic hypotension (nOH)].

[DISPLAY ONLY IF NOT OH/NOH DIAGNOSED BUT EXPERIENCE NOH SYMPTOMS]Throughout the remainder of the survey, we will use the term “nOH” when referring to your experience with the symptoms shown below that you experience when you sit up, stand up, are standing for long periods of time or have a change in position (i.e., sitting to standing). When we ask about symptoms in the survey, please only think about these symptoms. [DISPLAY SYMPTOMS EXPERIENCED EVERY TIME, DAILY, WEEKLY, MONTHLY TO A FEW TIMES A YEAR (Q120/SCALE 1-7,9) FROM Q120 IN ONE COLUMN BELOW]]

[CAREGIVER: [DISPLAY ONLY IF DIAGNOSED WITH OH OR NOH IN Q110] Throughout the remainder of the survey, we will focus on the experience of the person you care for with [IF FORMALLY DIAGNOSED WITH OH OR NOH IN Q110, DISPLAY: orthostatic hypotension (OH). In this survey, we will refer to it as nOH] [IF FORMALLY DIAGNOSED WITH OH NOH IN Q110, DISPLAY: neurogenic orthostatic hypotension (nOH)]. When we ask about symptoms, please only think about your loved one’s symptoms related to [IF FORMALLY DIAGNOSED WITH OH IN Q110, DISPLAY: orthostatic hypotension (OH)] [IF FORMALLY DIAGNOSED WITH NOH IN Q110, DISPLAY: neurogenic orthostatic hypotension (nOH)].

[CAREGIVER: DISPLAY ONLY IF NOT OH/NOH DIAGNOSED BUT EXPERIENCE NOH SYMPTOMS] Throughout the remainder of the survey, we will use the term “nOH” when talking about the symptoms shown below that the person you care for experiences when [IF Q111/1 DISPLAY: he IF Q111/2, DISPLAY she] sits up, stands up, are stands for long periods of time or has a change in position (i.e., sitting to standing). When we ask about symptoms in the survey, please only think about these symptoms. [DISPLAY SYMPTOMS EXPERIENCED EVERY TIME, DAILY, WEEKLY, MONTHLY TO A FEW TIMES A YEAR (Q120/SCALE 1-7,9) FROM Q120 IN ONE COLUMN BELOW]]

ALL CAREGIVERS

Q105a Overall, how would you describe the general health of the person you care for?

1. Poor
2. Fair
3. Good
4. Excellent

ALL QUALIFIED RESPONDENTS

Q205 [PATIENT: When did you first experience the symptoms below?]

[CAREGIVER: When did the person you care for first experience the symptoms below?

[DISPLAY DROPDOWN MENU FOR YEAR, RANGE: YEAR OF BIRTH – 2016]

ALL QUALIFIED RESPONDENTS

Q210 [PATIENT: Overall, how would you describe the severity of your nOH symptoms in the past month?]

[CAREGIVER: Overall, how would you describe the severity of the nOH symptoms of the person you care for in the past month?]

[PATIENT: My nOH symptoms are…]

[CAREGIVER: His/Her nOH symptoms are…]

1. Very mild
2. Mild
3. Moderate
4. Severe
5. Very severe

ALL QUALIFIED RESPONDENTS

Q215 [PATIENT: Overall, how severe were your nOH symptoms in the past month compared to 1 year ago?]

[CAREGIVER: Overall, how severe were the nOH symptoms of the person you care for in the past month compared to 1 year ago?]

[PATIENT: Compared to 1 year ago, my nOH symptoms are now…]

[CAREGIVER: Compared to 1 year ago, his/her nOH symptoms are now…]

1. Much less severe
2. Less severe
3. Unchanged
4. More severe
5. Much more severe

ALL QUALIFIED RESPONDENTS

Q220 [PATIENT: During what part of the day are your nOH symptoms most severe?]

[CAREGIVER: During what part of the day are the nOH symptoms of the person you care for most severe?]

[SINGLE RESPONSE]

1. [PATIENT: First thing in the morning (after I wake up and get out of bed)]

[CAREGIVER: First thing in the morning (after they wake up and get out of bed]

1. After a meal
2. Getting out of bed to use the bathroom at night
3. Other part of the day not mentioned above
4. [PATIENT: My symptoms are the same severity throughout the day]

[CAREGIVER: His/Her symptoms are the same severity throughout the day]

ALL QUALIFIED RESPONDENTS

Q222 [PATIENT: During what part of the day are your nOH symptoms most frequent?]

[CAREGIVER: During what part of the day are the nOH symptoms of the person you care for most frequent?]

[SINGLE RESPONSE]

1. [PATIENT: First thing in the morning (after I wake up and get out of bed)]

[CAREGIVER: First thing in the morning (after they wake up and get out of bed)]

1. After a meal
2. Getting out of bed to use the bathroom at night
3. Other part of the day not mentioned above
4. [PATIENT: My symptoms are the same frequency throughout the day]

[CAREGIVER: His/Her symptoms are the same frequency throughout the day]

ALL QUALIFIED RESPONDENT

Q225 [PATIENT: How often do any of your nOH symptoms get better or go away when you lie down or return to your original position (i.e., standing back to sitting)?]

[CAREGIVER: How often do any of the nOH symptoms of the person you care for get better or go away when he/she lies down or returns to his/her original position (i.e., standing back to sitting)?]

1. Never
2. Some of the time
3. Most of the time
4. Every time

ALL QUALIFIED RESPONDENTS

Q230 [PATIENT: In the past 12 months, how many times have you fallen because of your nOH symptoms? Please enter 0 in all boxes if you have not fallen at all in the past 12 months.

When answering about your falls, please only think of falls related to nOH (i.e. that occur when you sit up, stand up, are standing for long periods of time or have a change in position (i.e., sitting to standing)).

[CAREGIVER: In the past 12 months, how many times has the person you care for fallen because of his/her nOH symptoms? Please enter 0 in all boxes if he/she has not fallen at all in the past 12 months.

When answering about their falls, please only think of falls related to nOH (i.e. that occur when they sit up, stand up, are standing for long periods of time or have a change in position (i.e., sitting to standing)).

[PN: RANGE 0-99]

[RANDOMIZE 1-2]

1. [INSERT NUMERIC TEXT BOX] Falls in the past 12 months that required medical services but not hospitalization, such as a visit with a nurse or primary care physician, or emergency room visit
2. [INSERT NUMERIC TEXT BOX] Falls in the past 12 months that required hospitalization
3. [INSERT NUMERIC TEXT BOX] Falls in the past 12 months that did not require any medical services

ALL QUALIFIED RESPONDENTS

Q235 [PATIENT: How much do you agree or disagree with the following statements about your nOH symptoms?]

[CAREGIVER: How much do you agree or disagree with the following statements about the nOH symptoms of the person you care for?]

1. Strongly disagree
2. Somewhat disagree
3. Somewhat agree
4. Strongly agree
5. [PATIENT: I have a clear understanding about the cause of my nOH symptoms.]

[CAREGIVER:

1. [PATIENT: I wish I knew more about nOH symptoms.]

[CAREGIVER: The person I care for wishes he/she knew more about his/her nOH symptoms.]

1. [PATIENT: My nOH symptoms are more troublesome than my motor symptoms of PD (ie tremor, slowness of movement, rigidity, etc.). [DISPLAY ONLY FOR THOSE THAT HAVE BEEN DIAGNOSED WITH PD]]

[CAREGIVER: His/Her nOH symptoms are more troublesome than his/her motor symptoms of PD (i.e., tremor, slowness of movement, rigidity, etc.). [DISPLAY ONLY FOR THOSE THAT HAVE BEEN DIAGNOSED WITH PD]

1. [PATIENT: My nOH symptoms are more troublesome than my other symptoms caused by MSA (i.e., constipation, erectile dysfunction, urinary problems, trouble walking, etc.) [DISPLAY ONLY FOR THOSE THAT HAVE BEEN DIAGNOSED WITH MSA]]

[CAREGIVER: His/Her nOH symptoms are more troublesome than his/her other symptoms caused by MSA (i.e., constipation, erectile dysfunction, urinary problems, trouble walking, etc.) [DISPLAY ONLY FOR THOSE THAT HAVE BEEN DIAGNOSED WITH MSA]]

1. [PATIENT: I have trouble managing my nOH symptoms during the day.]

[CAREGIVER: The person I care for has trouble managing his/her nOH symptoms during the day.]

1. [PATIENT: My nOH symptoms appeared before I first developed motor symptoms of [DISPLAY “PD” or “MSA” DEPENDING ON THEIR EARLIER ANSWERS]. [DISPLAY ONLY FOR THOSE THAT HAVE BEEN DIAGNOSED WITH PD OR MSA]]

[CAREGIVER: His/Her nOH symptoms appeared before he/she first developed motor symptoms of [DISPLAY “PD” or “MSA” DEPENDING ON THEIR EARLIER ANSWERS]. [DISPLAY ONLY FOR THOSE THAT HAVE BEEN DIAGNOSED WITH PD OR MSA]]

1. [PATIENT: My nOH symptoms worsen in hot and/or humid conditions.]

[CAREGIVERS: His/Her nOH symptoms worsen in hot and/or humid conditions.]

1. [PATIENT: My nOH symptoms worsen after meals.]

[CAREGIVER: His/Her nOH symptoms worsen after eating a meal.]

Section 300: Functionality and Quality of Life

ALL QUALIFIED RESPONDENTS

Q300 [PATIENT: The next set of questions will focus on how your nOH symptoms impact your life.]

[CAREGIVER: The next set of questions will focus on how nOH symptoms impact the life of the person for whom you provide care.]

[PATIENT: Overall, how much of an impact do your nOH symptoms have on your life?]

[CAREGIVER: Overall, how much of an impact do his/her nOH symptoms have on his/her life?]

1. None
2. Very mild
3. Mild
4. Moderate
5. Severe
6. Very severe

ALL QUALIFIED RESPONDENTS

Q305 [PATIENTS: Overall, how much of an impact do your nOH symptoms have on your ability to perform everyday activities (e.g., housework, driving, getting dressed, exercising, participating in social activities)?]

[CAREGIVERS: Overall, how much of an impact do his/her nOH symptoms have on his/her ability to perform everyday activities (e.g., housework, driving, getting dressed, exercising, participating in social activities)?]

1. None
2. Very mild
3. Mild
4. Moderate
5. Severe
6. Very severe

ALL QUALIFIED RESPONDENTS

Q310 [PATIENT: In the past month, which of the following day-to-day activities, if any, has required assistance (from another person or device) as a result of your nOH symptoms? Please select all that apply.

Please do not include activities impacted by other conditions you may have, such as Parkinson’s disease (PD), multiple system atrophy (MSA), or pure autonomic failure (PAF). Please only think about required assistance due to your nOH symptoms.]

[CAREGIVER: In the past month, which of the following day-to-day activities, if any, has required assistance (from you, another person, or device) as a result of his/her nOH symptoms? Please select all that apply.

Please do not include activities impacted by other conditions he/she may have, such as Parkinson’s disease (PD), multiple system atrophy (MSA), or pure autonomic failure (PAF). Please only think about required assistance due to his/her nOH symptoms.]

MULTIPLE RESPONSE

1. Sitting up
2. Walking
3. Getting out of bed
4. Going up and down stairs
5. Getting out of a chair
6. Getting out of a car
7. Using the bathroom to urinate or defecate
8. Showering/bathing
9. Dressing/changing clothes
10. Doing household chores
11. Cooking/preparing food
12. Exercise/exertion
13. Some other daily activity/activities not listed above
14. [PATIENT: I did not require any assistance in the past month as a result of my nOH symptoms

[CAREGIVER: He/she did not require any assistance in the past month as a result of his/her nOH symptoms.

ALL QUALIFIED RESPONDENTS

Q315 [PATIENT: Which of the following activities have you had to reduce or stop completely as a result of your nOH symptoms? Please select all that apply.

Again, please do not include activities impacted by other conditions you may have, such as Parkinson’s disease (PD), multiple system atrophy (MSA), or pure autonomic failure (PAF). Please only think about changes you have made due to your nOH symptoms.]

[CAREGIVER: Which of the following activities have he/she had to reduce or stop completely as a result of his/her nOH symptoms? Please select all that apply.

Again, please do not include activities impacted by other conditions he/she may have, such as Parkinson’s disease (PD), multiple system atrophy (MSA), or pure autonomic failure (PAF). Please only think about changes he/she has made due to his/her nOH symptoms.]

1. Reduced
2. Stopped completely
3. No change
4. Not applicable
5. [MULTIPLE RESPONSE][WILL ONLY BE ASKED OF PATIENTS CURRENTLY EMPLOYED (Q715/ yes to any of codes 1-4 AND QP1/1) OR ALL QUALIFIED CAREGIVERS (QP1/2)] Number of work hours
6. Housework
7. Hobbies
8. Physical activity/exercise
9. Caring for loved ones (e.g., children, grandchildren)
10. Entertaining at home
11. Driving
12. Attending religious services/events
13. Time spent out of the house socializing (not including religious services)
14. Time spent out of the house on errands (i.e., not for pleasure)
15. Time spent with family and friends
16. [PATIENT: Another activity/Other activities not listed above as a result of my nOH symptoms]

[CAREGIVER: Another activity/Other activities not listed above as a result of his/her nOH symptoms]

1. [PATIENT: I have not made any changes as a result of my nOH symptoms]

[CAREGIVER: He/She has not made any changes as a result of his/her nOH symptoms

1. Travelling

ALL QUALIFIED PATIENTS [PATIENT QUESTION ONLY]

Q320 Which of the following act as a caregiver for you? Please select all that apply.

For example, a caregiver may be a spouse, family member, or friend who, without pay, accompanies you to physician visits, helps you make treatment decisions, helps you with daily household tasks, provides emotional support, etc.

A paid caregiver is someone who is paid to accompany you to physician visits, help you make treatment decisions, help you with daily household tasks, provides emotional support, etc.

[MULTIPLE RESPONSE]

1. Spouse or significant other
2. Child
3. Family member who is not a spouse or child
4. Friend
5. Volunteer
6. Paid caregiver (e.g. home health service)
7. Other
8. I do not have a caregiver

ALL QUALIFIED CAREGIVERS

Q320a Is there a paid caregiver that also supports the person you provide care for?

A paid caregiver is someone who is paid to accompany the person you care for to physician visits, help his/her make treatment decisions, help him/her with daily household tasks, provide emotional support, etc.

1. Yes
2. No

ALL QUALIFIED RESPONDENTS WHO HAVE A PAID CAREGIVER IN Q320/6 OR Q320a/1

Q325 [PATIENT: In the past month, on average, how many days each week were you visited by your paid caregiver and how many hours each week did they work?]

[CAREGIVER: In the past month, on average, how many days each week was the person you care for visited by his/her paid caregiver and how many hours each week did the paid caregiver work?]

Your best estimate is fine. Please round to the nearest whole number.

1. [NUMERIC TEXT BOX, RANGE 1-7] Average number of days visited each week
2. [NUMERIC TEXT BOX, RANGE 1-168] Average number of hours visited each week

ALL QUALIFIED RESPONDENTS WHO HAVE A CAREGIVER IN Q320 OR ANY CAREGIVER (Q320/NE8 OR QP1/2)

Q330 [PATIENTS: How much help do you need from your unpaid caregiver, paid caregiver, or unpaid or paid caregiver to perform daily activities?]

[CAREGIVERS: How much help does the person you provide care for need to perform daily activities?]

1. [PATIENT: I do not need help with any of my daily activities]

[CAREGIVER: He/She does not need help with any of his/her daily activities]

1. [PATIENT: I need help with some of my daily activities]

[CAREGIVER: He/She needs help with some of his/her daily activities]

1. [PATIENT: I need help with most of my daily activities]

[CAREGIVER: He/She needs help with most of his/her daily activities]

1. [PATIENT: I need help with all of my daily activities]

[CAREGIVER: He/She needs help with all of his/her daily activities]

1. [DISPLAY IF UNPAID CAREGIVER SELECTED AT Q320] [PATIENT: Unpaid caregiver]

[CAREGIVER: You]

1. [DISPLAY IF PAID CAREGIVER SELECTED AT Q320 OR Q320A] Paid/Professional caregiver

ALL QUALIFIED PATIENTS WHO DO NOT HAVE A CAREGIVER IN Q320 (Q320/8)

Q333 [PATIENTS: Regardless if you don’t have a caregiver, how much help do you need to perform daily activities?]

1. [PATIENT: I do not need help with any of my daily activities]
2. [PATIENT: I need help with some of my daily activities]
3. [PATIENT: I need help with most of my daily activities]
4. [PATIENT: I need help with all of my daily activities]

ALL QUALIFIED RESPONDENTS

Q335 [PATIENT: Thinking about the overall impact of your nOH symptoms on your life, how much do you agree or disagree with the following statements?]

[CAREGIVER: Thinking about the overall impact of nOH symptoms on the life of the person for whom you provide care, how much do you agree or disagree with the following statements?]

1. Strongly disagree
2. Somewhat disagree
3. Somewhat agree
4. Strongly agree
5. [PATIENT: My life has changed drastically because of my nOH symptoms.]

[CAREGIVER: His/Her life has changed drastically because of his/her nOH symptoms.]

1. [PATIENT: My quality of life has been negatively impacted by my nOH symptoms.]

[CAREGIVER: His/Her quality of life has been negatively impacted by his/her nOH symptoms.]

1. [PATIENT: My nOH symptoms have a minimal impact on my life.]

[CAREGIVER: His/Her nOH symptoms have a minimal impact on his/her life.]

1. [PATIENT: My nOH symptoms make every day a challenge for me.]

[CAREGIVER: His/Her nOH symptoms make every day a challenge for him/her]

1. [PATIENT: Despite my symptoms, I am able to lead a full and productive life.]

[CAREGIVER: Despite his/her symptoms, he/she is able to lead a full and productive life.]

1. [PATIENT: I have adequate support to help me cope with my symptoms.]

[CAREGIVER: He/she has adequate support to help him/her cope with his/her symptoms.]

1. [PATIENT: I often hide or minimize my nOH symptoms.]

[CAREGIVER: He/She often hides or minimizes his/her nOH symptoms.]

1. [PATIENT: I feel like a burden on my loved ones because of my nOH symptoms.]

[CAREGIVER: He/She feels like a burden on his/her loved ones because of his/her nOH symptoms.]

1. [PATIENT: I can live a full life with my nOH symptoms.]

[CAREGIVER: He/She can live a full life with his/her nOH symptoms.]

1. [PATIENT: Living with my nOH symptoms causes me a significant amount of anxiety or worry.]

[CAREGIVER: Living with his/her nOH symptoms causes him/her a significant amount of anxiety or worry.]

1. [PATIENT: In the past 12 months, managing my nOH symptoms have caused me to feel discouraged or depressed.

[CAREGIVER: In the past 12 months, managing his/her nOH symptoms have caused him/her to feel discouraged or depressed.]

1. [PATIENT: My nOH symptoms have robbed me of my independence.]

[CAREGIVER: His/Her nOH symptoms have robbed him/her of his/her independence.]

Section 400: Health Care Provider Interaction

ALL QUALIFIED RESPONDENTS

Q400 [PATIENT: For the next few questions, we will focus on your experience with the health care you receive. Which of the following describe the specialties of health care providers you regularly see to manage your [INSERT: (IF Q110/1 AND PERSONALLY DIAGNOSED) Parkinson’s disease (PD), (IF Q110/2 AND PERSONALLY DIAGNOSED) multiple system atrophy (MSA), or (IF Q110/3 AND PERSONALLY DIAGNOSED) pure autonomic failure (PAF)]?

Please include health care providers you visit regardless if they are managing your symptoms from nOH. Please select all that apply.]

[CAREGIVER: For the next few questions, we will focus on the experience of the person you care for with the health care they receive. Which of the following describe the specialties of the health care providers he/she regularly sees to manage his/her [INSERT: Parkinson’s disease (PD), multiple system atrophy (MSA), or pure autonomic failure (PAF) BASED ON ANSWER PROVIDED AT Q110]?

Please include health care provider he/she visits regardless if they are managing his/her symptoms from nOH. Please select all that apply.]

[MULTIPLE RESPONSE]

1. Autonomic specialist
2. General cardiologist
3. Electrophysiologist
4. Hypertension (blood pressure) specialist
5. Parkinson’s disease/movement disorder specialist
6. Nephrologist
7. General neurologist
8. Gerontologist
9. Primary care provider (internist, family practitioner, general practitioner)
10. Other specialty [ANCHOR]
11. None of these [EXCLUSIVE, ANCHOR]
12. Not sure/I don’t remember [EXCLUSIVE, ANCHOR]
13. Gastroenterologist
14. Otolaryngologist
15. Speech therapist
16. Podiatrist
17. Pain specialist

REPORTED BEING DIAGNOSED WITH OH OR NOH IN Q110

Q405

[PATIENT: What is the specialty of the health care provider who initially diagnosed you with orthostatic hypotension (OH) or neurogenic orthostatic hypotension (nOH), and the specialty of the health care provider primarily managing your OH or nOH?

What is the specialty of the health care provider who initially diagnosed his/her with orthostatic hypotension (OH)] or neurogenic orthostatic hypotension (nOH) and the specialty of the health care provider primarily managing his/her OH or nOH]?

[THIS WILL BE SET UP AS A GRID WHERE A RESPONDENT CAN SELECT A SPECIALTY FOR EACH OF THE OFFICE/CLINIC WHO DIAGNOSED AND SPECIALTY WHO IS CURRENTLY MANAGING]

1. Specialty of the health care provider who diagnosed
2. Specialty of the health care provider who is primarily managing OH/nOH symptoms
3. [SINGLE SELECT]Autonomic specialist
4. General cardiologist
5. Electrophysiologist
6. Hypertension (blood pressure) specialist
7. Parkinson’s disease/movement disorder specialist
8. Nephrologist
9. General neurologist
10. Gerontologist
11. Primary care clinician (internist, family practitioner, general practitioner)
12. Other specialty
13. None of these
14. [PATIENT: I am not currently seeing a clinician to manage my OH/nOH symptoms.]

[CAREGIVER: He/She is not currently seeing a clinician to manage his/her OH/nOH symptoms.]

1. Not sure/I don’t remember
2. Gastroenterologist
3. Otolaryngologist
4. Speech therapist
5. Podiatrist
6. Pain specialist

REPORTED BEING DIAGNOSED WITH OH OR NOH IN Q110

Q406

[PATIENT: How many health care providers did you see before being diagnosed with orthostatic hypotension (OH)] or neurogenic orthostatic hypotension (nOH)]? Your best estimate is fine.

[CAREGIVER: How many health care providers did he/she see before being diagnosed with orthostatic hypotension (OH)] or neurogenic orthostatic hypotension (nOH)]? Your best estimate is fine.

[NUMERIC TEXTBOX, RANGE 1-99] Number of health care providers seen before being formally diagnosed

ALL QUALIFIED RESPONDENTS

Q410 [PATIENT: Thinking back to when you first experienced any of your orthostatic hypotension (OH) or neurogenic orthostatic hypotension (nOH) symptoms, who initiated the first conversation about your symptoms with a health care provider?]

[CAREGIVER: Thinking back to when the person you care for first experienced any of his/her orthostatic hypotension (OH) or neurogenic orthostatic hypotension (nOH)]symptoms, who initiated the first conversation about his/her symptoms with a health care provider?]

1. [PATIENT: I initiated the conversation]

[CAREGIVERS: The person I care for initiated the conversation]

1. [IF HAVE A CAREGIVER (Q320/NE 8][PATIENT: My caregiver initiated the conversation

[CAREGIVERS: I initiated the conversation]

6 [IF HAVE A CAREGIVER (Q320/NE 8][PATIENT: We both initiated the conversation

7 [CAREGIVERS: We both initiated the conversation]

1. A health care provider initiated the conversation
2. Not sure/I cannot recall who initiated the conversation
3. [PATIENT: I have not discussed my nOH symptoms with any care provider]

[CAREGIVER: The person I care for has not discussed his/her nOH symptoms with any care provider]

DISCUSSED SYMPTOMS WITH PROVIDER AT Q410 (Q410/1-4, 6-7) AND (Q120/1,3-14,15= Q121/1-7, 9)

Q415 [PATIENT: You mentioned you talked to a provider about your nOH symptoms. Which nOH symptoms specifically have you discussed with a health care provider? Please select all that apply.]

[CAREGIVER: You mentioned the you or the person you care for talked to a provider about his/her nOH symptoms. Which nOH symptoms specifically has he/she discussed with a health care provider? Please select all that apply.]

[MULTIPLE RESPONSE]

1. Dizziness or lightheadedness [DISPLAY IF Q120/1 AND Q121/1-7,9]
2. Feeling faint [DISPLAY IF Q120/15 AND Q121/1-7,9]
3. Passing out or fainting [DISPLAY IF Q120/3 AND Q121/1-4]
4. Falling [DISPLAY IF Q120/4 AND Q121/1-7,9]
5. Blurry vision [DISPLAY IF Q120/6 AND Q121/1-7,9]
6. Tunnel vision [DISPLAY IF Q120/7 AND Q121/1-7,9]
7. Fatigue when standing [DISPLAY IF Q120/8 AND Q121/1-7,9]
8. Pain running down neck and across your shoulders [DISPLAY IF Q120/9 AND Q121/1-7,9]
9. [BLANK]
10. Nausea [DISPLAY IF Q120/10 AND Q121/1-7,9]
11. Difficulty breathing [DISPLAY IF Q120/11 AND Q121/1-7,9]
12. Difficulty walking (e.g., Feeling like their legs will buckle beneath them) [DISPLAY IF Q120/12 AND Q121/1-7,9]
13. Confused, foggy, inability to think clearly [DISPLAY IF Q120/13 AND Q121/1-7,9]
14. Blood pressure drops when you stand [DISPLAY IF Q120/14 AND Q121/1-7,9]
15. Other
16. [PATIENT: Not sure/I cannot recall any of the symptoms I have discussed with a health care provider]

[CAREGIVER: Not sure/I cannot recall any of the symptoms he/she has discussed with a health care provider.]

DISCUSSED SYMPTOMS WITH PROVIDER AT Q410 (Q410/1-4, 6,7)

Q420 [PATIENT: How much time passed between when you first experienced your symptoms from orthostatic hypotension (OH) or neurogenic orthostatic hypotension (nOH), and when you first discussed them with a health care provider? Your best estimate is fine.]

[CAREGIVER: How much time passed between when the person you care for first experienced he/she symptoms from orthostatic hypotension (OH) or neurogenic orthostatic hypotension (nOH), and when he/she first discussed them with a health care provider? Your best estimate is fine.]

1. Less than 1 month
2. 1 – 6 months
3. Between 6 – 12 months
4. More than a year
5. Not sure/I don’t remember

DISCUSSED SYMPTOMS WITH PROVIDER AT Q410 (Q410/1-4,6,7)

Q425 [PATIENT: What was the outcome of the first discussion with your health care provider about your nOH symptoms? Please select all that apply.]

[CAREGIVER: What was the outcome of the first discussion with his/her health care provider about his/her nOH symptoms? Please select all that apply.]

[MULTIPLE RESPONSE]

1. [PATIENT: I was given a diagnosis]

[CAREGIVER: He/She was given a diagnosis]

1. [PATIENT: I was instructed to take additional blood pressure recordings at home]

[CAREGIVER: He/She was instructed to take additional blood pressure recordings at home]

1. [PATIENT: I was scheduled for additional tests

[CAREGIVER: He/She was scheduled for additional tests]

1. [PATIENT: I was referred to another physician]

[CAREGIVER: He/She was referred to another physician]

1. [PATIENT: I was provided guidance on how to manage my symptoms

[CAREGIVER: He/She was provided guidance on how to manage his/her symptoms]

[PATIENT: I was told to drink more water and add more salt to my diet]

[CAREGIVER: He/She was told to drink more water and add more salt to his/her diet.]

1. A medication(s) was prescribed
2. Other
3. [PATIENT: Nothing was done to address my symptoms after I first discussed them with my health care provider]

[CAREGIVER: Nothing was done to address his/her symptoms after I first discussed them with his/her health care provider]

1. Not sure/I don’t remember

ALL QUALIFIED RESPONDENTS

Q430 [PATIENT: How many times in the past 12 months have you visited a health care provider to manage your nOH symptoms? Please provide your best estimate.]

[CAREGIVERS: How many times in the past 12 months has the person you care for visited a health care provider to manage his/her nOH symptoms? Please provide your best estimate.]

[NUMERIC TEXTBOX. RANGE 0-365] visits in the past 12 months

PATIENT WITH A CAREGIVER HAS VISITED HEALTH CARE PROVIDER AT LEAST ONCE IN LAST 12 MONTHS (Q430/1-365)

Q433 [PATIENT: What percent of the time does your caregiver accompany you to your visits to your health care providers? Please consider all of your health care visits, not only those related to managing your nOH symptoms. Please provide your best estimate.]

[CAREGIVERS: What percent of the time do you accompany the person you care for to their visits to their health care providers? Please consider all of the health care visits for the person you care for, not only those related to managing their nOH symptoms. Please provide your best estimate.]

[NUMERIC TEXTBOX. RANGE 0-100]

ALL QUALIFIED RESPONDENTS

Q435 [PATIENT: We will now move on to specific ways your nOH symptoms are checked and monitored.]

[CAREGIVER: We will now move on to specific ways the nOH symptoms of the person you care for are checked and monitored.]

[PATIENT: In what position(s) is your blood pressure measured in the health care provider’s office?]

[CAREGIVERS: In what position(s) is his/her blood pressure measured in the health care provider’s office?]

[MULTIPLE RESPONSE]

1. Measured while sitting down only
2. Measured while lying down only
3. Measured while standing only
4. Measured when sitting or lying down, and then measured again while standing
5. Not sure/I don’t remember
6. Not checked during office visit

ALL QUALIFIED RESPONDENTS

Q440 [PATIENT: In what position(s) is your blood pressure measured at home?]

[CAREGIVER: In what position(s) is his/her blood pressure measured at home?]

1. Measured while sitting down only
2. Measured while lying down only
3. Measured while standing only
4. Measured when sitting or lying down, and then measured again while standing
5. Not sure/I don’t remember
6. [PATIENT: My blood pressure is not measured at home]

[CAREGIVER: His/Her blood pressure is not measured at home]

ALL QUALIFIED RESPONDENTS WHO MEASURE BLOOD PRESSURE AT HOME (Q440/1-4)

Q442 [PATIENT: During what time of day did your physician ask you to check your blood pressure?

[CAREGIVER: During what time of day does he/she ask you to check their blood pressure?]

[MULTIPLE RESPONSE]

Morning

After meals

At night

1. No specific time is given
2. Not sure/I don’t remember

ALL QUALIFIED RESPONDENTS WHO MEASURE BLOOD PRESSURE AT HOME (Q440/1-4)

Q444 [PATIENT: Do you routinely experience a rise in blood pressure when you lie down at night.]

[CAREGIVER: Does the person I care for routinely experiences a rise in his/her blood pressure when he/she lies down at night.]

1. Yes
2. No
3. [PATIENT: My blood pressure is not measured to identify if it routinely rises when I lie down at night]
   [CAREGIVER: His/Her blood pressure is not measured to identify if it routinely rises when he/she lies.]
4. I don’t know

ALL QUALIFIED RESPONDENTS

Q445 [PATIENT: Does your health care provider ask you to monitor changes in your nOH symptoms between visits?]

[CAREGIVERS: Does his/her health care provider ask him/her to monitor changes in his/her nOH symptoms between visits?]

[RANDOMIZE]

1. [PATIENTS: Yes, my health care provider asks me to monitor changes in my nOH symptoms and to report them at the next visit]

[CAREGIVERS: Yes, his/her health care provider asks that his/her monitor changes in his/her nOH symptoms monitored and to report them at the next visit]

1. [PATIENTS: Yes, my health care provider asks me to monitor changes in my nOH symptoms and report them in between visits]

[CAREGIVERS: Yes, his/her health care provider asks that his/her monitor changes in his/her nOH symptoms and report them in between visits]

1. [PATIENTS: No, my health care provider does not ask me to monitor or communicate changes in my nOH symptoms. ]

[CAREGIVERS: No, his/her health care provider does not ask him/her to monitor or communicate changes in his/her nOH symptoms.]

1. Not sure/I don’t remember

ALL QUALIFIED RESPONDENTS

Q446 [PATIENT: Has your health care provider primarily treating your nOH told you that your symptoms are caused by the fact that your body is not releasing enough norepinephrine.]

[CAREGIVER: Has his/her health care provider primarily treating his/her nOH told him/her that his/her symptoms are caused by the fact that his/her body is not releasing norepinephrine properly.]

1. Yes
2. No
3. I don’t recall

ALL QUALIFIED RESPONDENTS

Q450 [PATIENTS: Thinking about your communication with the health care provider who primarily manages your [FOR THOSE WHO DO NOT HAVE A HCP CURRENTLY MANAGING THEIR SYMPTOMS AT Q405 (Q405/12 AND SPECIALTY OF HEALTH CARE PROVIDER WHO IS CURRENTLY MANAGING NOH SYMPTOMS), INSERT RESPONSE AT Q110/1-5 AND “nOH” FOR ALL OTHERS], how much do you agree or disagree with the following statements?]

[CAREGIVERS: Thinking about [IF Q111/1 DISPLAY: his IF Q111/2, DISPLAY her] communication with the health care provider who primarily manages his/her [FOR THOSE WHO DO NOT HAVE A HCP CURRENTLY MANAGING THEIR SYMPTOMS AT Q405, INSERT RESPONSE AT Q110/1-5 AND “nOH” FOR ALL OTHERS], how much do you agree or disagree with the following statements?]

1. Strongly disagree
2. Somewhat disagree
3. Somewhat agree
4. Strongly agree
5. Not sure/don't know
6. [PATIENTS: I am satisfied with the quality of communication with my health care provider.]

[CAREGIVERS: He/She is satisfied with the quality of communication with his/her health care provider.]

1. [PATIENTS: I believe my health care provider understands the impact of my nOH symptoms on my quality of life.]

[CAREGIVERS: He/She feels his/her health care provider understands the impact of his/her nOH symptoms on his/her quality of life.]

1. [PATIENTS: I am uncomfortable talking to my health care provider about the impact of nOH symptoms on my life.]

[CAREGIVERS: He/She is uncomfortable talking to his/her health care provider about the impact of nOH symptoms on his/her life.]

1. [PATIENTS: I typically do not bring up my nOH symptoms to my health care provider unless they are severe.]

[CAREGIVERS: He/She typically does not bring up his/her nOH symptoms to his/her health care provider unless they are severe.]

1. [PATIENTS: I have to mention nOH symptoms repeatedly in order for my health care provide to take them seriously.]

[CAREGIVERS: He/She feels he/she has to mention nOH symptoms repeatedly in order for his/her health care provide to take them seriously.]

1. [PATIENTS: The path to a nOH diagnosis was very frustrating for me.]

[CAREGIVERS: The path to a nOH diagnosis was very frustrating for the person I care for.]

[PATIENTS: My healthcare provider and I have tried several medications and we can’t get my nOH symptoms under control.]

[CAREGIVERS: His/Her healthcare provider and the person I care for have tried several medications and they can’t get his/her nOH symptoms under control.]

1. [PATIENTS: My health care provider has been able to provide me with a solution to better manage my nOH symptoms.]

[CAREGIVERS: He/She feels health care provider has been able to provide him/her with a solution to better manage his/her nOH symptoms.]

Section 500: Symptom Management and Treatments/Interventions

ALL QUALIFIED RESPONDENTS

Q500 [PATIENTS: Thinking about your symptoms from orthostatic hypotension (OH) or neurogenic orthostatic hypotension (nOH), overall, has your health care provider who manages your symptoms counseled you to do any of the following to manage your nOH symptoms? Please select all that apply.]

[CAREGIVERS: Thinking about his/her symptoms from orthostatic hypotension (OH) or neurogenic orthostatic hypotension (nOH), overall, has his/her health care provider who manages his/her symptoms counseled him/her to do any of the following to manage his/her nOH symptoms? Please select all that apply.]

[MULTIPLE RESPONSE]

1. Increase salt
2. Wear compression stockings and/or abdominal binders
3. Elevate the head of the bed
4. Adjust or discontinue other blood pressure or heart medications
5. [PATIENT: Adjust your Parkinson's disease (PD) medications]

[CAREGIVER: Adjust their Parkinson's disease (PD) medications]

Physical counter maneuvers (e.g., standing up and crossing legs, standing up and squeezing hands tightly)

Avoiding quick positional changes

Avoiding heated environments

1. Another action not listed above
2. [PATIENT: I have not been counseled to do anything to manage my symptoms]

[CAREGIVER: The person I care for has not been counseled to do anything to manage their symptoms.]

1. Not sure/I don’t remember
2. Increase fluid intake

ALL QUALIFIED RESPONDENTS

Q505 [PATIENTS: Overall, how well do you think your nOH symptoms are being managed?]

[CAREGIVERS: Overall, how well do you think his/her nOH symptoms are being managed?]

1. Not at all well
2. Not very well
3. Somewhat well
4. Very well

ALL QUALIFIED RESPONDENTS

Q510 [PATIENT: Have you ever been prescribed a medication to manage your nOH symptoms?]

[CAREGIVERS: Has the person you care for ever been prescribed a medication to manage his/her nOH symptoms?]

1. Yes
2. No
3. Not sure/Don’t know

HAVE BEEN PRESCRIBED A TREATMENT AT Q500 OR MEDICATION AT Q510 (Q500/1-9, 12 OR Q510/1)

Q515 [PATIENTS: Overall, how well do you think your nOH symptoms are being managed by the treatment(s) or medication(s) you have been prescribed?]

[CAREGIVERS: Overall, how well do you think his/her nOH symptoms are being managed by the treatment(s) or medication(s) he/she has been prescribed?]

1. Not at all well
2. Not very well
3. Somewhat well
4. Very well

ALL QUALIFIED RESPONDENTS

Q525 [PATIENTS: How much do you agree or disagree with the following statements about your nOH symptoms?]

[CAREGIVERS: How much do you agree or disagree with the following statements about his/her nOH symptoms?]

1. Strongly disagree
2. Somewhat disagree
3. Somewhat agree
4. Strongly agree
5. [PATIENTS: I wish I had more resources to help me manage my nOH symptoms.]
6. [PATIENTS: Management of my nOH symptoms has improved since diagnosis.]

[CAREGIVERS: The person I care for feels management of his/her nOH symptoms has improved since diagnosis.]

1. [PATIENTS: There are treatments and medications available to effectively manage nOH symptoms.]

[CAREGIVERS: The person I care for feels there are treatments and medications available to effectively manage nOH symptoms.]

1. [PATIENTS: I really struggle to get my nOH symptoms under control.]

[CAREGIVERS: The person I care for really struggles to get his/her nOH symptoms under control.]

1. [PATIENTS: The number of treatments and medications for my nOH symptoms is burdensome.]

[CAREGIVER: The person I care for feels the number of treatments and medications for his/her nOH symptoms is burdensome.]

1. [PATIENTS: I have found the right health care provider to effectively manage my nOH symptoms.]

[CAREGIVERS: The person I care for believes he/she has found the right health care provider.]

1. [PATIENTS: The path to finding the right health care provider to help me manage my nOH symptoms has been difficult.]

[CAREGIVERS: The person I care for believes that the path to finding the right health care provider has been difficult.]

Section 600: Caregiver Experience

ALL QUALIFIED CAREGIVERS

Q600 The next set of questions will focus on how nOH symptoms of the person for whom you provide care impact your life.

Overall, how much of an impact does the nOH symptoms of the person for whom you provide care have on your life?

1. None
2. Mild
3. Moderate
4. Severe
5. Very severe

ALL QUALIFIED CAREGIVERS

Q605 How much do you agree or disagree with the following statements about the impact of caring for someone with nOH symptoms?

1. Strongly disagree
2. Somewhat disagree
3. Somewhat agree
4. Strongly agree
5. Not applicable [ONLY SHOW FOR CODE 9]
6. My life has changed dramatically because of his/her symptoms related to nOH.
7. My quality of life has been negatively impacted by his/her nOH symptoms.
8. His/Her nOH symptoms have a minimal impact on my life.
9. ] His/Her nOH symptoms make every day a challenge for me.
10. Despite his/her symptoms, I am able to lead a full and productive life.
11. I have adequate support to help me cope with his/her symptoms.
12. Dealing with his/her nOH symptoms causes me a significant amount of anxiety.
13. Helping him/her managing his/her nOH symptoms have caused me to feel depressed.
14. I have had to reduce my work hours or stop work completely in order to provide care for my loved one.
15. Through caring for someone of nOH symptoms I feel I have lost my sense of self.
16. Being a caregiver of someone with nOH symptoms makes me feel isolated.
17. Monitoring his/her changes in blood pressure is burdensome.

ALL QUALIFIED CAREGIVERS

Q610 Thinking about your experience, how much do you agree or disagree with the following statements about the care provided for your loved one?

1. Strongly disagree
2. Somewhat disagree
3. Somewhat agree
4. Strongly agree
5. I am satisfied with the quality of my communications with his/her health care provider.
6. I believe his/her health care provider understands the impact of nOH symptoms on his/her quality of life.
7. I am uncomfortable talking to his/her health care provider about the impact of nOH symptoms on his/her life.
8. I typically do not bring up his/her nOH symptoms to the health care provider unless they are severe.
9. I have to mention nOH symptoms repeatedly in order for his/her health care provider to take them seriously.
10. The path to a nOH diagnosis was very frustrating for me.
11. health care provider has been able to provide him/her with an effective solution(s) to manage his/her nOH symptoms.
12. I am satisfied with the quality of care provided by his/her health care provider.
13. I feel comfortable talking with his/her health care providers about the impact of nOH symptoms on my life as a caregiver.

ALL QUALIFIED CAREGIVERS

Q615 How much do you agree or disagree with the following statements about your loved one’s nOH symptoms?

1. Strongly disagree
2. Somewhat disagree
3. Somewhat agree
4. Strongly agree

[RANDOMIZE]

1. I wish I had more resources to help me manage his/her nOH symptoms.
2. His/Her nOH symptoms are being effectively managed.
3. There are treatments and medications available to effectively manage his/her nOH symptoms.
4. I feel we have found the right health care provider to effectively manage his/her nOH symptoms.
5. The path to finding the right health care provider has been difficult.
6. I have a clear understanding about what is causing his/her nOH symptoms.
7. I wish I knew more about his/her nOH symptoms.

Section 700: Demographics

ALL RESPONDENTS

Q740 [PATIENTS: Have you ever been diagnosed with any of the following conditions? Please select all that apply.]

[CAREGIVERS: Has the person for whom you provide care ever been diagnosed with any of the following conditions? Please select all that apply.]

[MULTIPLE RESPONSE]

1. Peripheral neuropathy
2. Anxiety
3. Depression
4. Cancer
5. Diabetes
6. Thyroid disease
7. High blood pressure
8. Heart condition (e.g., atrial fibrillation (AFib), heart attack)
9. Stroke
10. Chronic Obstructive Pulmonary Disease (COPD)
11. Sleep disorders (e.g., insomnia, sleep apnea, restless legs syndrome)
12. REM sleep behavior disorder (acting out your dreams)
13. None of these
14. Not sure
15. Decline to answer

ALL RESPONDENTS

Q745 [PATIENT: Which type of health plan or health insurance do you have? Please select all that apply.]

[CAREGIVER: Which type of health plan or health insurance does the person you care for have? Please select all that apply.]

[MULTIPLE RESPONSE] [5 cannot be selected with 7; 6 cannot be selected with 1, 2, 3, OR 4; 9 CANNOT BE SELECTED WITH ANY OTHER OPTION]

1 Health care through a job or union

2 Health care through spouse/partner’s job or union

3 Health care through the individual market (individual, family, or small business) but not through healthcare.gov or a state-based exchange (for example, through a private insurance company, an online insurance seller, or an agent or broker)

4 Health care through healthcare.gov or a state-based exchange

5 Medicare or a Medicare HMO (a government plan that pays health care bills for people age 65 and older and for some people with disabilities)

6 Medicaid, a Medicaid HMO, Medi-Cal or public aid (government plans that pay health care bills for people who meet certain financial rules)

7 Veteran Affairs (VA) benefits from my own service or through my spouse

8 Some other type of health plan or coverage

9 [PATIENT: I don’t have health insurance]

[CAREGIVER: The person I care for does not have health insurance.]

10 Decline to answer

ALL RESPONDENTS [WILL BE ASKED IN STANDARD DEMOGRAPHIC SET UP]

Q750 Which of the following income categories best describes your total 2015 household income before taxes?

1. Less than $15,000
2. $15,000 to $24,999
3. $25,000 to $34,999
4. $35,000 to $49,999
5. $50,000 to $74,999
6. $75,000 to $99,999
7. $100,000 to $124,999
8. $125,000 to $149,999
9. $150,000 to $199,999
10. $200,000 to $249,999
11. $250,000 or more
12. Decline to answer

ALL RESPONDENTS

Q760 What is the highest level of education you have completed or the highest degree you have received?

1. Less than high school
2. Completed some high school
3. Completed high school
4. Job-specific training program(s) after high school
5. Some college, but no degree
6. Associate Degree
7. College (such as B.A., B.S.)
8. Some graduate school, but no degree
9. Graduate degree (such as MBA, MS, MD, PhD)

ALL RESPONDENTS

Q770 What is your marital status?

1. Never married
2. Married or civil union
3. Divorced
4. Separated
5. Widow/Widower
6. Living with partner

ALL RESPONDENTS

Q775 [PATIENT: Which of the following best describes your current living situation?]

[CAREGIVER: Which of the following best describes current living situation for the person for whom you provide care?]

1. My own residence
2. Relative’s residence
3. Assisted-living facility
4. Nursing home
5. Other
